# Application of Fuzzy Delphi in the Selection of COPD Risk Factors among Steel Industry Workers

**Published:** 2017

**Authors:** Rahmat Dapari, Halim Ismail, Rosnah Ismail, Noor Hassim Ismail

**Affiliations:** Community Health Department, National University of Malaysia, Malaysia.

**Keywords:** Fuzzy Delphi method, Chronic Obstructive Pulmonary Disease, Risk factors, Expert judgment

## Abstract

**Background:**

The Delphi method has been widely applied in many study areas to systematically gather experts’ input on particular topic. Recently, it has become increasingly well known in health related research. This paper applied the Fuzzy Delphi method to enhance the validation of a questionnaire pertaining chronic obstructive pulmonary disease (COPD) risk factors among metal industry workers.

**Materials and Methods:**

A detailed, predefined list of possible risk factors for COPD among metal industry workers was created through a comprehensive and exhaustive review of literature from 1995 to 2015. The COPD questionnaire were distributed among people identified as occupational, environmental, and hygiene experts. Linguistic variable using Likert scale was used by the expert to indicate their expert judgment of each item. Subsequently, the linguistic variable was converted into a triangular fuzzy number. The average score of the fuzzy number will be used to determine whether the item will be removed or retained.

**Results:**

Ten experts were involved in evaluating 26 items. The experts were in agreement with most of the items, with an average fuzzy number range between 0.429 and 0.800. Two items were removed and three items were added, leaving a total 26 items selected for the COPD risk factors questionnaire. The experts were in disagreement with each other for items F10 and F11 where most of the experts claimed that the question is too subjective and based on self-perception only.

**Conclusion:**

The fuzzy Delphi method enhanced the accuracy of the questionnaire pertaining to COPD risk factors, and decreased the length of the established tools.

## INTRODUCTION

The Delphi method was introduced in the 1950s in defense research, which was followed by application in societal, transportation, environmental, science, and technological research. It has become a fundamental tool for those in the area of technological forecasting and is used today in many technologically oriented corporations (1). Recently, The Delphi method has been widely applied for systematically gathering experts’ input on a particular topic, especially in health related research, as this method is particularly well suited to health issues (2). The Delphi method has been used in occupational health research (3). Subsequently the Delphi research method has become widely used in healthcare research (4).

The Delphi method is an iterative process for collecting and distilling anonymous experts’ judgments using a series of data collection and analysis techniques interspersed with a feedback mechanism (5). The Delphi method has undergone steady development and modification since its inception in the 1950s (2). Over the years, many labels describing types of Delphi have been used. Some labels relate to the type of application, some to the method of scoring used, and some just imply a difference in approach (6). The fuzzy Delphi method is one example from numerous Delphi methods that have undergone modification and development. The fuzzy Delphi method is a combination of the traditional Delphi method and Fuzzy Set Theory, which aims to address some of the ambiguity of the expert panel consensus. It is a more advanced version of the Delphi method in that it utilizes triangulation statistics to determine the distance between the levels of consensus within the expert panel (7). Furthermore, the objective of using Delphi is to achieve group consensus (6).

The definition of “expert” in the Delphi method varies according to the context and field of interest. Being an expert entails the acquisition of experience, a special skill in, or knowledge on a particular subject. However, the experts selected do not necessarily need to have standard academic qualification such as a class honors degree or PhD (8). There are four requirements for expertise. The first component is knowledge and practical engagement with the issue under investigation. The Second component is the capacity and willingness of the experts to participate. The third component is having sufficient time to be dedicated to the Delphi exercise, and the last component is effective communication between the researcher and the experts (8). In general, an expert could be anyone with relevant input. Some applications require panels covering a wide range of interests and disciplinary viewpoints (1). However, since expert opinion is sought, a purposive sample may be necessary. It may begin with the researcher seeking help from a supervisor to identify the initial group of experts, followed by using a “snowball” sampling technique to generate a subsequent expert panel (5). Fortunately, in many health-related problems, the identity of these experts is commonly acknowledged within the circle of health professionals and the Delphi panel can be recruited swiftly and without controversy (2). Although it is clear that the selected experts are multifaceted, there will continue to be difficulties in defining and justifying their selection (9). In fact, currently there is no exact criterion listed in the literature concerning the selection of Delphi experts (10). However, it becomes the responsibility of each researcher to choose the most appropriate group of experts and to defend that choice (11).

Apart from the selection of experts, there are a few other components that influence the decision to use the fuzzy Delphi method in this study. The size of the expert panel in the Delphi method will vary, but with a homogenous group of experts, good result can be obtained even with small panels of 10–15 individuals (8). Nowadays, there are many different modes of interaction available and, with the advent of e-mail, pen and paper-based Delphi’s are less common.

The most significant benefit of e-mail is the expediency provided by this mode of interaction. Quick responses can be obtained and the raw data is already in a digital format, which eliminates the tedious task of transcription (5). Lastly, feedback is an important feature of the Delphi and most feedback is numerical or statistical with some form of aggregated group response (6). This paper aims to display an application of the fuzzy Delphi method in selection of COPD risk factors among metal industry workers to ensure the usefulness of each item is useful before the questionnaire is distributed in other larger study populations.

COPD is a common preventable and treatable disease characterized by persistent airflow limitation that is usually progressive and associated with an enhanced chronic inflammatory response in the airways (12). COPD is a complex, multifactorial, and progressive disease and is now known to be the most frequent chronic disease in developing country workers (13). In Malaysia, the prevalence of moderate to severe COPD in persons 30 years and older is 4.7% (14). Occupational exposures is one the factors associated with COPD (15), for example exposure to dusts, noxious gases/vapors, fumes (16), and metal dust (17). Smelters and furnace workers have the highest prevalence of COPD followed by casters and other professional groups (13). Throughout the world, many people suffer from COPD for many years, and die prematurely from it or its associated complications (12). The disease is causing rising economic burden (18), especially in developing countries (19).

## MATERIALS AND METHODS

A detailed list of possible risk factors for COPD among metal industry workers was developed through a comprehensive and exhaustive literature review. We searched the National Library of Medicine (MEDLINE) database, Google scholar (Advanced Google search), and the WHO Library and Information Networks for Knowledge Database (WHOLIS) using the search terms “COPD and epidemiology”, “COPD and risk factors”, “COPD and occupation”, and “COPD and metal dust”. We focused on articles published in the past 20 years in order to gain as many relevant risk factors related to COPD. Snowball searching was employed based on reference lists of identified publications. Only papers published in English were included.

**Figure 1.**
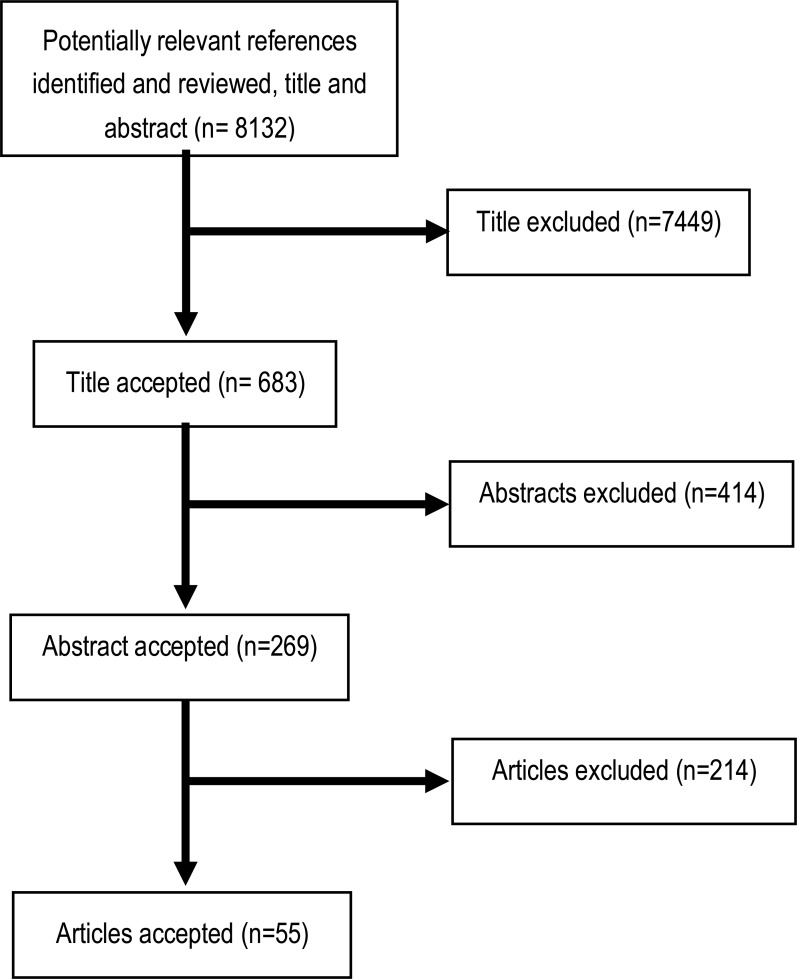
Occupational and non-occupational related risk factors for COPD

The initial search identified 8132 articles of potential interest. The number of articles decreased to 683 after duplicates and irrelevant titles were removed. Based on titles, the remaining articles were identified as potentially relevant for the purpose of this study. However, 414 articles were removed after reviewing the abstract, as they were found to be irrelevant. A total of 269 articles remained for full review, but 214 were excluded as either the full article was not available (not free access) or it did not fulfill the inclusion and exclusion criteria. Thus, the final 55 articles were accepted for data extraction (Figure 1).

A list of possible risk factors were group according to possible domains. Each of the items was phrased as a question. As a result, a hybrid COPD risk factors questionnaire was established consisting of 23 items requiring a quantitative response and 5 items requiring a qualitative response. Four experts were involved in the first round of application of the fuzzy Delphi method. Subsequently, three additional items were added. Ten experts were involved in the second round of the application of the fuzzy Delphi method and no further items were added.

The sample consisted of ten experts consist of four academics (two from the occupational field and two environmentalists), two registered occupational health doctors (one from the private sector and one steel industries panel doctor), one registered hygienist, and three public health specialists with working experience involved in publishing research in occupational and environment related issues. Experts were selected based on their knowledge and at least five years of experience (in the field or in research) in the occupational area.

The fuzzy Delphi method was used for systematically gathering input from relevant experts. Expert panels were informed either face to face between the researcher and the expert, or via phone or e-mail. The study objectives were explained to ensure that the expert understood the justification for the study, the purpose of employing fuzzy Delphi, and it methodology. The fuzzy Delphi technique was explained in detail as well as how to score each item. The risk factor COPD questionnaire consists of 25 items measured using a 5-point Likert scale (1 = strongly disagree, 2 = disagree, 3 = neutral, 4 = agree, 5 = strongly agree). Experts were required to indicate the extent of their agreement with the question statements given. Experts could also be asked to give an explanation or justification for their response and to add an item if required.

### Data Analysis

The fuzzy Delphi method comprises two integral elements. First, each expert’s responses are converted into triangular fuzzy numbers to identify their level of agreement for each item. Next, the defuzzification process was conducted to determine the value reflecting the degree of consensus of the respondents. The Likert scale is helpful in the development of a linguistic variable. The linguistic variables are then converted into triangular fuzzy numbers. The scaled used in this study can be seen in Table 1. The ranking for each variable according to the experts’ judgment was then ascertained through the process of defuzzification, to determine an average score of fuzzy numbers using following formula:

Xmax = ((X1+X2+X3)/3)/N = α

X: Item
Xmax: average score of fuzzy number
N: number of expert panel involved
(X1, X2, X3): triangular fuzzy number according to linguistic variable

**Table 1.** Likert scale, linguistic variable and triangular fuzzy number

**Likert scale**	**Linguistic variable**	**Triangular fuzzy number**
1	Strongly disagree	0.0, 0.0, 0.2
2	Disagree	0.0, 0.2, 0.4
3	Neutral	0.2, 0.4, 0.6
4	Agree	0.4, 0.6, 0.8
5	Strongly agree	0.6, 0.8, 1.0

This study chose an *α*-cuts = 0.5 as the threshold to select the item. The value of 0.5 was the mid-point (median) of the interval [0, 1]. This threshold value has been used in previous studies and has been used as a reference in the introduction of basic fuzzy Delphi methodology. During the first cycle, the experts may provide additional items if they believe that the item is necessary to attain the study objective. Then, the item will be included for the next round and the expert panel will give a score. The cycle will be repeated until there is no more additional input from the expert panel.

### Ethics

These studies were reviewed and approved by ethics committee of the National University of Malaysia (FF-2015-318).

## RESULTS

Analysis of the experts’ judgment using the Fuzzy Delphi method yielded the following findings (Table 2). The experts were in agreement with most of the items with an average fuzzy number range between 0.429 and 0.800. Two items were removed and three items were added leaving a total of 24 items, which were selected for the COPD risk factors questionnaire. The experts were in disagreement with each other about items F10 an F11, with most of the experts maintaining that the question is too subjective and based on self-perception only.

**Table 2.** Risk factors for COPD questionnaire

**Item**	**Content of risk factor**	**Average score of fuzzy number Xmax)**
F1	Smoking status	0.571
F2	Duration of smoking in years	0.638
F3	Average of cigarette smoked per day	0.686
F4	Age start smoking	0.714
F5	History of smoking if currently not smoking	0.657
F6	Duration stop smoking in years	0.686
F7	Employment history (duration, work unit)	0.629
F8	Exposure to metal dust on each work unit	0.686
F9	Dust deposited on skin and clothing	0.629
F10	Can you smell something while exposed to dust	0.486
F11	Does your friend smell something while exposed to dust	0.429
F12	PPE compliant on respirator/mask/	0.771
F13	Smoking at workplace (active smoker)	0.800
F14	Exposed to smoke at workplace (passive smoker)	0.800
F15	Having meal and drink at workplace other than restroom	0.629
F16	Shower at workplace before going home	0.686
F17	Change clothing before going home	0.771
F18	Employment history other than metal industry	0.800
F19	Part time work that exposed to metal dust and VGDF	0.667
F20	Hobby that exposed to metal dust and VGDF	0.800
F21	Personnel medical history (severe pulmonary infection, TB)	0.800
F22	Family medical history (COPD)	0.800
F23	Workplace exposure assessment	0.800
F24	Work process changes	0.629
F25	Production changes	0.657
F26	Control measure changes	0.657

## DISCUSSION

The purpose of this study was to examine the extent of experts’ consensus on the risk factors of COPD among metal industry workers. The experts were in disagreement with each other regarding whether the workers or coworkers can smell something while being exposed to metal dust (see items F10 and F11). As the score of these items were below 0.6, items F10 and F11 were excluded from the COPD risk factor questionnaire. Item F10 asks, “Can you smell something while you are exposed to metal dust?” and item F11 asks, “Do any of your friend ever complain to you that they smell something while being exposed to metal dust?” The final scores were 0.486 and 0.429, respectively. This indicates that the expert panels believed that the questions were too subjective, depending on the workers’ perception. Therefore, the response from the respondent would not help to establish whether they had been exposed to metal dust.

### Strengths and limitations

The strengths of this study include a comprehensive literature review of COPD related risk factor in occupational and steel industries setting. Second, experts were well informed regarding the objectives and how the fuzzy Delphi method was used. Thirdly, anonymity in the fuzzy Delphi method removes the effects of status and group pressure, and prevents domination by a small group that can arise in face-to-face group meetings. This could occur if the panels contain a dominant senior member who believes that they are the most expert, has a powerful personality, and is outspoken, as these types of people tend to influence the non-dominant panel members to agree with the other experts’ opinions. Therefore, the fuzzy Delphi method allows experts to provide an honest expression of their views. Fourth, statistical aggregation of group responses allows for a quantitative analysis and interpretation of data. The ability to use statistical analysis techniques will further reduce the potential of group pressure for conformity. Thus, the consensus of an expert panel is made based on quantitative analyses of each item in the COPD risk factors questionnaire. These methods also shorten the questionnaire and are cheaper compared to other methods such as the “Focus Group Discussion”. The limitations of this study include the difference in the years of experience and positions of each expert, which might contribute to variation in their understanding about COPD risk factors. Thus, the differences in knowledge, qualifications, working experience, and research experience among several experts could influence the accuracy of their judgment of each item. Arguments could also be made that an expert who is in the public sector will hold differing opinions from one in the private sector or university sector. However, public health specialists in an occupational related task, hygienists in the private sector, an occupational health doctors in the private sector, panel occupational doctors in metal industries, and university lecturers might all have multifaceted perspectives on the relevant risk factors for COPD among metal industry workers.

### Recommendation

Selecting experts who have had significant experience with the intervention approach and having a balanced number of experts with similar job scope will help to improve the validity of these findings. This research study may elicit more related studies on the application of the fuzzy Delphi method to evaluate and establish questionnaires on health related issues. The fuzzy Delphi method has been, and will continue to be, an important data collection methodology with a wide variety of applications and uses for people who want to gather information from experts in a particular subject.

## CONCLUSION

The application the fuzzy Delphi method by conducting an expert survey and analyzing the results in selection COPD risk factors helps to enhance the validity and shorten the duration of established tools. The result from this study will serve as a foundation for the construction of further content validation through pre-tests and pilot studies.

## References

[1] Linstone HA Turoff M. The Delphi Method. Techniques and applications. 2002;53.

[2] de Meyrick J. The Delphi method and health research. Health education. 2003;103(1):7–16.

[3] van der Beek AJ Frings-Dresen MH van Dijk FJ Houtman IL. Priorities in occupational health research: a Delphi study in The Netherlands. Occup Environ Med 1997;54(7):504–10. 9282128 10.1136/oem.54.7.504 PMC1128821

[4] Keeney S Hasson F McKenna H. Consulting the oracle: ten lessons from using the Delphi technique in nursing research. J Adv Nurs 2006;53(2):205–12. 16422719 10.1111/j.1365-2648.2006.03716.x

[5] Skulmoski GJ Hartman FT Krahn J. The Delphi method for graduate research. Journal of information technology education 2007;6:1.

[6] Mullen PM. Delphi: myths and reality. J Health Organ Manag 2003;17(1):37–52. 12800279 10.1108/14777260310469319

[7] Ishikawa A Amagasa M Shiga T Tomizawa G Tatsuta R Mieno H. The max-min Delphi method and fuzzy Delphi method via fuzzy integration. Fuzzy sets and systems 1993;55(3):241–53.

[8] Adler M Ziglio E. Gazing into the oracle: The Delphi method and its application to social policy and public health. Jessica Kingsley Publishers; 1996.

[9] Baker J Lovell K Harris N. How expert are the experts? An exploration of the concept of ‘expert’ within Delphi panel techniques. Nurse Res 2006;14(1):59–70. 17100214 10.7748/nr2006.10.14.1.59.c6010

[10] Hsu CC Sandford BA. The Delphi technique: making sense of consensus. Practical assessment, research & evaluation 2007;12(10):1–8.

[11] Sumsion T. The Delphi technique: an adaptive research tool. British Journal of Occupational Therapy 1998;61(4):153–6.

[12] Rabe KF Hurd S Anzueto A Barnes PJ Buist SA Calverley P Fukuchi Y Jenkins C Rodriguez-Roisin R Van Weel C Zielinski J. Global strategy for the diagnosis, management, and prevention of chronic obstructive pulmonary disease: GOLD executive summary. American journal of respiratory and critical care medicine 2007;176(6):532–55. 17507545 10.1164/rccm.200703-456SO

[13] Bala S Tabaku A. Chronic obstructive pulmonary disease in iron-steel and ferrochrome industry workers. Cent Eur J Public Health 2010;18(2):93–8. 20939259 10.21101/cejph.a3548

[14] Regional COPD Working Group COPD prevalence in 12 Asia-Pacific countries and regions: projections based on the COPD prevalence estimation model. Respirology 2003;8(2):192–8. 12753535 10.1046/j.1440-1843.2003.00460.x

[15] Govender N Lalloo UG Naidoo RN. Occupational exposures and chronic obstructive pulmonary disease: a hospital based case-control study. Thorax 2011;66(7):597–601. 21502099 10.1136/thx.2010.149468

[16] Graziani M Doney B Hnizdo E Villnave J Breen V Weinmann S Assessment of lifetime occupational exposure in an epidemiologic study of COPD. The Open Epi J 2012;5:27–35.

[17] Weinmann S Vollmer WM Breen V Heumann M Hnizdo E Villnave J COPD and occupational exposures: a case-control study. J Occup Environ Med 2008;50(5):561–9. 18469625 10.1097/JOM.0b013e3181651556

[18] Guarascio AJ Ray SM Finch CK Self TH. The clinical and economic burden of chronic obstructive pulmonary disease in the USA. Clinicoecon Outcomes Res 2013;5:235–45. 23818799 10.2147/CEOR.S34321 PMC3694800

[19] Salvi SS Barnes PJ. Chronic obstructive pulmonary disease in non-smokers. Lancet 2009;374(9691):733–43. 19716966 10.1016/S0140-6736(09)61303-9

